# Nitrogen addition, rather than altered precipitation, stimulates nitrous oxide emissions in an alpine steppe

**DOI:** 10.1002/ece3.8196

**Published:** 2021-10-10

**Authors:** Yang Yang, Yuanming Xiao, Changbin Li, Bo Wang, Yongheng Gao, Guoying Zhou

**Affiliations:** ^1^ Northwest Institute of Plateau Biology Chinese Academy of Science Xining China; ^2^ University of Chinese Academy of Science Beijing China; ^3^ Key Laboratory of Tibetan Medicine Research Chinese Academy of Sciences Xining China; ^4^ College of Agriculture and Animal Husbandry Qinghai University Xining China; ^5^ Institute of Mountain Hazards and Environment Chinese Academy of Science Chengdu China

**Keywords:** climate change, functional gene, greenhouse gas, nitrification, Qinghai–Tibetan Plateau

## Abstract

Anthropogenic‐driven global change, including changes in atmospheric nitrogen (N) deposition and precipitation patterns, is dramatically altering N cycling in soil. How long‐term N deposition, precipitation changes, and their interaction influence nitrous oxide (N_2_O) emissions remains unknown, especially in the alpine steppes of the Qinghai–Tibetan Plateau (QTP). To fill this knowledge gap, a platform of N addition (10 g m^−2^ year^−1^) and altered precipitation (±50% precipitation) experiments was established in an alpine steppe of the QTP in 2013. Long‐term N addition significantly increased N_2_O emissions. However, neither long‐term alterations in precipitation nor the co‐occurrence of N addition and altered precipitation significantly affected N_2_O emissions. These unexpected findings indicate that N_2_O emissions are particularly susceptible to N deposition in the alpine steppes. Our results further indicated that both biotic and abiotic properties had significant effects on N_2_O emissions. N_2_O emissions occurred mainly due to nitrification, which was dominated by ammonia‐oxidizing bacteria, rather than ammonia‐oxidizing archaea. Furthermore, the alterations in belowground biomass and soil temperature induced by N addition modulated N_2_O emissions. Overall, this study provides pivotal insights to aid the prediction of future responses of N_2_O emissions to long‐term N deposition and precipitation changes in alpine ecosystems. The underlying microbial pathway and key predictors of N_2_O emissions identified in this study may also be used for future global‐scale model studies.

## Graphical abstract

Our field experiments show that the alpine steppe was a net source of N2O. Our results also demonstrate that N addition intensified N2O emission, while altered precipitation and its interaction with N addition did not affect N2O emission.

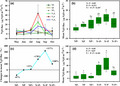

## 1 INTRODUCTION

Nitrous oxide (N_2_O), a non‐carbon dioxide (CO_2_) greenhouse gas, has a global warming potential nearly 300‐fold greater than that of CO_2_ over a 100‐year lifespan (Dijkstra et al., 2013). The accumulation of N_2_O in the atmosphere will deplete stratospheric ozone and contribute to global warming (Ravishankara et al., 2009). The main sources of atmospheric N_2_O are closely associated with soil nitrogen (N) cycling (i.e., nitrification and denitrification) of terrestrial ecosystems, which contribute to ~56%–70% of global N_2_O emissions (Butterbach‐Bahl et al., 2013). As the main component of terrestrial ecosystems, grasslands are one of the most widely distributed vegetation types on earth (Scurlock et al., 2002). On the Qinghai–Tibetan Plateau (QTP), alpine grassland ecosystems (e.g., alpine meadows and alpine steppes) are huge nitrogen (N) reservoirs because of sluggish microbial decomposition (Kou et al., 2019). However, the substantial labile N stored in alpine soils, which is a large source of N_2_O, is often neglected (Mao et al., 2020). Global change, particularly atmospheric N deposition and changing precipitation regimes, has considerable consequences for storage and patterns of N in alpine ecosystems (Fu and Shen, 2017; Lin et al., 2016). Given that alpine grasslands may possess the capacity for N_2_O release and are sensitive to global change (Xiao et al., 2020), understanding how alpine soil N_2_O emissions respond to N deposition and precipitation changes is crucial for predicting future atmospheric N_2_O concentrations.

The main regulatory factors for plant communities and soil ecological processes in grasslands are N and water. Field simulations of the impact of atmospheric N deposition on N_2_O emissions are not scarce, especially in the alpine grasslands of the QTP. However, reports of the effects of N addition in these ecosystems are inconsistent. N addition has been shown to significantly increase soil N_2_O emissions, because N input elevates the concentration of inorganic N and the abundance of functional microbes in the soil (Geng et al., 2019; Peng et al., 2018; Wu et al., 2020; Yan et al., 2018). In addition, a greater labile carbon (C) supply (e.g., litter decomposition or root exudation) under N enrichment provides substrate C for heterotrophic denitrifiers, thereby stimulating N_2_O emissions (Brown et al., 2012; Dijkstra et al., 2013). However, Zhu et al. (2015) showed that N input did not affect N_2_O emissions. A possible interpretation of this finding is that low temperature and inadequate soil moisture limit the activities of microorganisms associated with N cycling in cold conditions (Banerjee et al., 2016; Curtis et al., 2006; Schaufler et al., 2010). Despite this work on grasslands, the response of N_2_O emissions to long‐term N deposition on the QTP remains understudied.

Soil N_2_O emissions are also susceptible to hydrologic variations (Knapp et al., 2002). Generally, changes in soil water content influence N mineralization and organic matter degradation, which then affect the provision of N and C reactants for N cycling processes. On a global scale, elevated precipitation in grassland ecosystems accelerates N_2_O emissions while decreased precipitation mitigates N_2_O emissions. These processes are predominantly regulated by shifts in soil water availability (Li et al., 2020). By contrast, Liu et al. (2014) showed that short‐term water increment did not affect N_2_O emissions from semi‐arid steppes. Even increased precipitation decreased N_2_O emissions in arid grasslands (Cai et al., 2016). This finding may be attributable to soil leaching and run‐off events caused by the increased rainfall, which intensified the loss of inorganic N in soil and thereby limited soil N cycling (Cregger et al., 2014). Little is known about how long‐term precipitation changes impact N_2_O emissions on the QTP. Both N and water affect soil biogeochemical cycles. N deposition and variation in precipitation usually occur simultaneously; thus, their effects are interdependent (Harpole et al., 2007). The combined effect of N deposition and altered precipitation on N_2_O emissions is also unknown. N cycling microbiomes play a crucial role in regulating soil N dynamics and global climate stabilization. On the QTP, it is also unclear how pivotal N cycling functional microorganisms respond to global change and which microbes better explain N_2_O emissions.

Due to multifactorial climate change and intensive interventions targeting anthropogenic activities, the environmental conditions of the QTP have undergone dramatic changes in the past few decades (Gong et al., 2017). The amount, frequency, and intensity of precipitation increased from the year 1975 to 2014 (Ge et al., 2017). The QTP also experienced pronounced N deposition during the period 1990–2003, with an average of 7.3 N kg ha^−1^ year^−1^ (Lü and Tian, 2007). The alpine steppes, the largest grassland ecosystem on the QTP, are extremely sensitive to global change (Ding et al., 2016; Wang et al., 2011). Therefore, understanding the effects of N enhancement and altered precipitation on N_2_O emissions in the alpine steppes is essential. This study consists of altered precipitation and N addition manipulation experiments that were conducted in an alpine steppe on the QTP in 2013. We monitored the N_2_O flux during the 2020 growing season (May to October) based on in situ experiments. To identify the key abiotic and biotic factors regulating N_2_O emissions, we measured N_2_O flux on six consecutive days in mid‐August (during peak plant growth). Soils were also collected to measure abiotic parameters and functional microbes, including nitrifiers (ammonia‐oxidizing bacteria: AOB; ammonia‐oxidizing archaea: AOA) and denitrifiers (*nirS*‐, *nirK*‐, and *nosZ* gene‐containing microorganisms). The objectives of the study were to (1) assess whether N_2_O emissions were altered by long‐term N addition, precipitation changes, and their interaction and (2) identify the mechanisms that regulated N_2_O emissions under N addition and altered precipitation patterns.

## 2 MATERIALS AND METHODS

### 2.1 Site description and experimental design

The study area is a typical alpine steppe, which is situated in the northeastern Qinghai–Tibetan Plateau (QTP), China (37°18′N, 100°15′E). The study site exhibits a plateau continental climate. The average annual temperature is ~0.1℃. The mean annual precipitation is ~390 mm, most of which occurs from June to August. The vegetation is mainly dominated by grasses, such as *Stipa purpurea* Grisebach, *Leymus secalinus* (Georgi) Tzvel, and *Poa crymophila* Keng (Xiao et al., 2020). The growing season is from May to October and peaks in August. At the beginning of the experiments, the chemical properties of the soil (0–30 cm depth) were as follows: total N, 2.5 g/kg; NO_3_^−^‐N, 11.5 mg/kg; NH_4_^+^‐N, 5.1 mg/kg. The topsoil is defined as a Haplic Calcisol (62% sand, 33% silt, and 5% clay) according to the FAO soil classification system, with a pH of 8.3.

**FIGURE 1:**
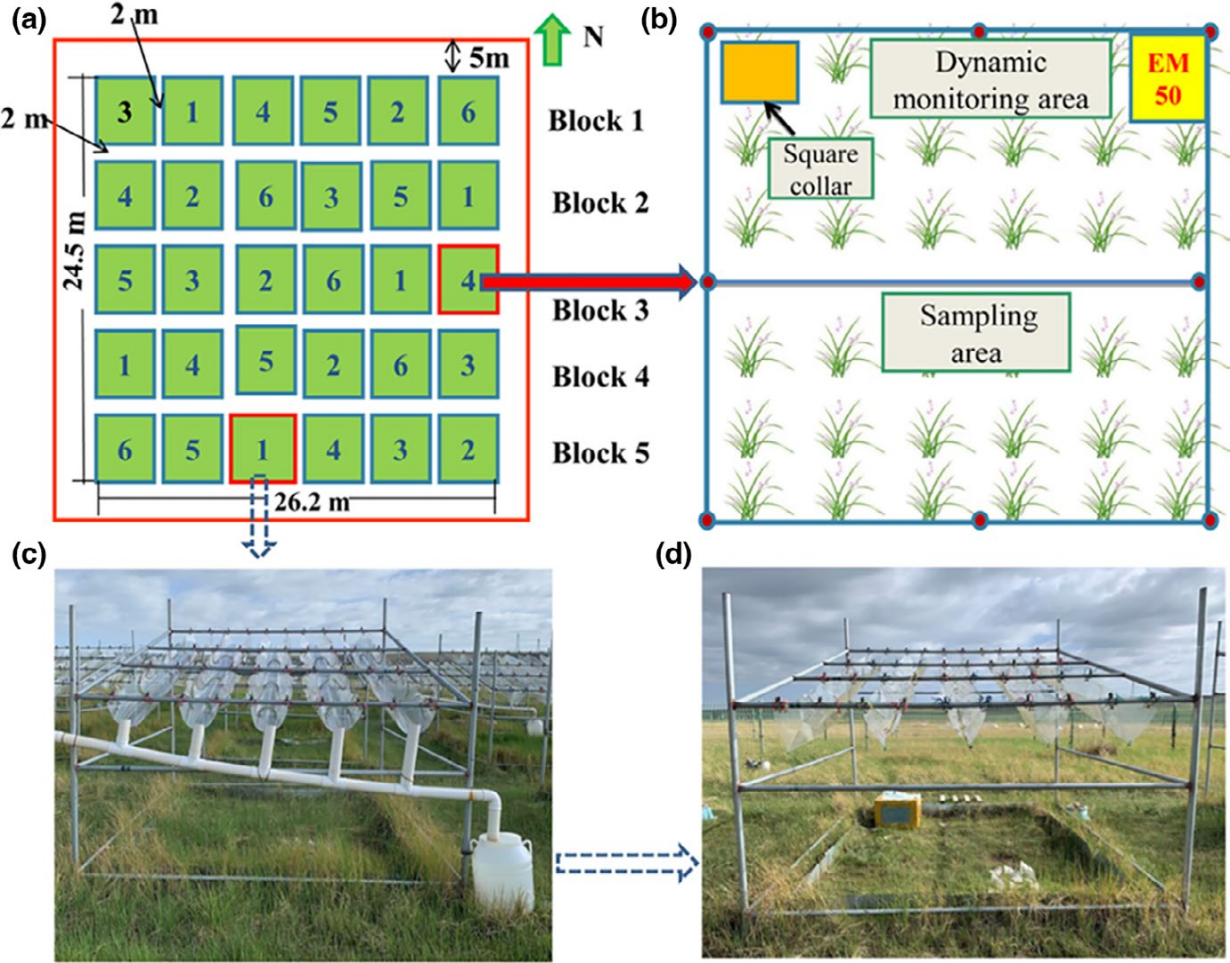
Platform for nitrogen addition and altered precipitation experiments. (a) and (b): Experimental treatments: 1, NP−; 2, NP (CK); 3, NP+; 4, N+P−; 5, N+P; 6, N+P+. N: ambient N deposition; P: ambient precipitation; N+: N addition; P−: 50% reduced precipitation; P+: 50% increased precipitation. (c): 50% reduced precipitation treatment. (d): 50% increased precipitation treatment

The experimental platform was established in 2013 (Figure 1). The experiments consisted of six different treatments (NP: ambient nitrogen with ambient precipitation; NP−: ambient nitrogen with 50% reduced precipitation; NP+: ambient nitrogen with 50% increased precipitation; N + P: nitrogen addition with ambient precipitation; N + P−: nitrogen addition with 50% reduced precipitation; and N + P+: nitrogen addition with 50% increased precipitation). These 30 plots (2.7 m × 3.3 m each) were randomly established in a 5×6 block design and were each separated by a buffer zone (2 m wide) (Figure 1a). As the experimental plot was relatively small (Figure 1c,d), it was divided into a sampling area (mainly used for soil and plant sampling) and a dynamic monitoring area (mainly used for N_2_O flux monitoring and plant community investigations) to ensure its integrity and continuity (Figure 1b). We calculated the precipitation treatments based on the area of the experimental plot. The upper part of the experimental plot was divided into two equal parts, and polyvinyl chloride boards without slots were then installed at equal distances to reduce ambient precipitation by 50% (Figure 1c). The collected water (50% ambient precipitation) was immediately transferred (by sprinkling evenly) to the 50% increased precipitation treatment section after the rain (Figure 1d). To avoid light differences between the treatment plots, the same PVC boards with slots were also installed on the ambient precipitation and 50% precipitation increment treatment plots. N fertilizer (NH_4_NO_3_: 10 g m^−2^ year^−1^) was dissolved in 1 L water and was evenly applied two times to the N supply plots (June and July every year). Identical amounts of water without N fertilizer were sprayed evenly on the ambient treatment plots. A previous study indicated that the N saturation level was 8 g m^−2^ year^−1^ in this study area (Peng et al., 2017). Therefore, the current N input level (10 g m^−2^ year^−1^) should be sufficient to simulate N saturation of the alpine grasslands.

### 2.2 N_2_O flux measurements

In 2013, a 40 cm × 40 cm square stainless‐steel collar was permanently inserted into the topsoil (~10 cm), which located in the dynamic monitoring area of each plot (Figure 1b). The in situ N_2_O flux was measured using static chamber with insulation materials and gas chromatography techniques. During gas collection (between 8 am and 12 noon), a chamber (30 cm tall) with an electric fan (to mix the air) was placed on the collar. Gas samples (100 ml) were collected by medical syringes at intervals of 0, 10, 20, and 30 min and then promptly injected into multi‐layer foil sampling bags (Delin Inc., Dalian, China). In 2020, we collected gas samples three times per month (May–October). Furthermore, we conducted that gas sample collection during six consecutive days in mid‐August (plant growth peak). The collected gas samples were immediately transferred to the laboratory and then determined for N_2_O concentration using a GC‐7890B gas chromatograph (Agilent Technologies Limited Co., Chengdu, China). While collecting gas samples (plant growth peak), the soil volumetric water content (VWC) and temperature at the depth of 10 cm were measured in each plot adjacent to the collar using a hand‐held moisture probe and a digital thermometer, respectively. The N_2_O flux was calculated as follows:

$$F=\rho \times \frac{V}{A}\times \frac{T_{0}}{T}\times \frac{P}{P_{0}}\times \frac{dc}{dt}$$

where *F* is the N_2_O flux (μg N_2_O m^−2^ h^−1^); *ρ* is the standard status N_2_O density (1.97 kg m^3^); *V* is the volume of the static chamber (m^3^); and *A* is the base area of the static chamber (m^2^). *T*_0_ and *T* are the standard temperature (273 K) and the static chamber temperature (K), respectively. *P*_0_ and *P* are the standard pressure (1013 hPa) and the air pressure (hPa), respectively. The rate of increase in the N_2_O concentration in the static chamber (10^−6^ h^−1^) is d*c*/d*t*.

### 2.3 Soil and plant sampling and chemical analyses

To identify the mechanisms regulating N_2_O flux responses to N input and altered precipitation, plant and soil samples were collected at the peak of plant growth (because N_2_O emissions mainly occurred at this period). First, three 25 cm × 25 cm quadrants were randomly placed in each plot, and then, all living plants were clipped as aboveground biomass. After removal of the aboveground plants, three root cores (internal diameter 8 cm and depth 10 cm) were collected and then mixed. The mixed root cores were washed with water in a 0.4‐mm sieve. The live roots were selected by their color, texture, and incidental fine roots (Peng et al., 2018) and were used as belowground biomass. The collected aboveground and belowground biomasses were oven‐dried at 60℃ to a constant mass and then weighed. Three more soil cores (internal diameter 3 cm and depth 10 cm) were collected near each collar (for a total of 90 soil cores) and were then homogenized to acquire one compound sample (for a total of 30 soil samples). The collected soil samples were separated into three subsamples by a sieve (2 mm). The first subsample was immediately preserved at −80℃ for DNA extraction and also analysis of the abundances of key microbial functional genes. The second subsample was stored at 4℃ to determine the soil ammonium (NH_4_^+^‐N) and nitrate (NO_3_^−^‐N) concentrations. The third subsample was air‐dried to determine the soil pH. The available N (NH_4_^+^‐N and NO_3_^−^‐N) concentrations in soil were determined using a flow injection analyzer (Autoanalyzer 3 SEAL, Bran and Luebbe, Norderstedt, Germany) after extracting fresh soil with 1 M KCl solution. The pH of the air‐dried soil was measured using a pH electrode (soil‐to‐deionized water ratio of 1:2.5).

### 2.4 Soil DNA extraction and real‐time quantitative PCR (qPCR)

Soil DNA was extracted from 0.5 g frozen soil using a kit (E.Z.N.A.^®^ DNA Kit, Omega Bio‐Tek, Norcross, GA, USA) based on the manufacturer's instructions. The DNA extract was checked on 1% agarose gel. The quality of the DNA was evaluated with a NanoDrop 2000 UV‐vis spectrophotometer (Thermo Scientific, Wilmington, DE, USA). The nitrification‐related *amoA* gene in ammonia‐oxidizing bacteria (AOB) and archaea (AOA) was determined. The *nirS*, *nirK*, and *nos*Z genes, which are associated with denitrification, were also determined in denitrifying microorganisms. The functional gene copy numbers were amplified using an ABI 7300 Real‐Time PCR System (ABI, CA, U.S.A.). PCRs were performed in triplicate. The PCR mixtures contained 10 μl 2X ChamQ SYBR Color qPCR Master Mix, 0.8 μl forward primer 5 (μM), 0.8 μl reverse primer (5 μM), 2 μl template DNA, 0.4 μl 50 X ROX Reference Dye 1, and 6 μl ddH_2_O. The functional genes, primers, and sequences used for PCRs are summarized in Table 1. The standard curve of each amplified gene was constructed using a 10‐fold dilution of plasmid DNA (containing the target gene). The PCR efficiency was between 89% and 101%; the *R* ^2^ ranged from 0.98 to 0.99.

**TABLE 1:** The targeted genes, primers pairs, and thermal cycling conditions for PCRs

Target gene	Primer name	Sequence (5′–3′)	Product size (bp)	Thermal profile
Archaeal amoA	Arch‐amoAF	STAATGGTCTGGCTTAGACG	635	30 s at 95℃, followed by 40 cycles of 10 s at 95℃
	Arch‐amoAR	GCGGCCATCCATCTGTATGT		30 s at 55℃, and 1 min at 72℃
Bacterial amoA	bamoA1F	GGGGTTTCTACTGGTGGT	491	1 min at 95℃, followed by 40 cycles of 30 s at 94℃,
	bamoA2R	CCCCTCKGSAAAGCCTTCTTC		30 s at 57℃, and 1 min at 72℃
nirS	cd3aF	GTSAACGTSAAGGARACSGG	425	2 min at 95℃, followed by 38 cycles of 10 s at 95℃
	R3cdR	GASTTCGGRTGSGTCTTGA		30 s at 55℃, and 1 min at 72℃
nirK	FlaCuF	ATCATGGTSCTGCCGCG	471	1 min 30 s at 95℃, followed by 38 cycles of 10 s at 95℃
	R3CuR	GCCTCGATCAGRTTGTGGTT		30 s at 58℃, and 40 s at 72℃
nosZ	CHEND‐nosZ‐1126F	GGGCTBGGGCCRTTGCA	255	30 s at 95℃, followed by 40 cycles of 10 s at 95℃
	CHEND‐nosZ‐1381R	GAAGCGRTCCTTSGARAACTTG		30 s at 60℃, and 1 min at 72℃

### 2.5 Statistical analyses

Before statistical analysis, we examined whether the data conformed to a normal distribution (Shapiro–Wilk test) and tested for homogeneity of variance (Levene's test). We conducted data analysis according to the following five steps. First, a Two‐way analysis of variance (ANOVA) was used to examine the effects of N addition, altered precipitation, and their interaction on the following: soil abiotic parameters (soil temperature, moisture, pH, NH_4_^+^‐N, and NO_3_^−^‐N); plant properties (aboveground and belowground biomass); N_2_O flux (emission peak); and the functional gene abundance related to nitrification (AOA, AOB) and denitrification (*nirS*, *nirK*, and *nos*Z). Second, a repeated‐measures ANOVA was performed to assess the effects of treatments on N_2_O flux during the growing season. Significant differences of the above‐mentioned parameters were assessed using post hoc tests (Duncan's test at *p* < .05). Third, multiple regression was used to explore the factors that significantly affect N_2_O flux in biotic (plant properties and functional genes) and abiotic (soil physicochemical properties) parameters. Fourth, variation partitioning analysis (VPA) was performed to evaluate the contribution of biotic and abiotic factors to the variation in N_2_O flux. Finally, structural equation model (SEM) analysis was performed to investigate the direct and indirect effects of biotic and abiotic factors on N_2_O flux. These statistical analyses were carried out in SPSS version 21.0 (SPSS, Chicago, IL, U.S.A.) and were visualized using Sigmaplot 12.5 software (Systat Software Corporation, U.S.A.).

## 3 RESULTS

### 3.1 Soil environment factors and plant properties

The soil abiotic parameters and plant attributes significantly differed by treatment (Table 2). N addition significantly reduced soil pH but had no effect on soil temperature and humidity. In contrast, N input significantly increased available N concentrations in soil and aboveground and belowground biomasses. Increased precipitation significantly increased soil moisture and aboveground biomass. Precipitation reduction significantly reduced soil moisture and soil NH_4_^+^‐N. However, soil pH, NO_3_^−^‐N, and belowground biomass were not affected by precipitation changes. Except for soil temperature, the combination of N addition and precipitation changes did not affect other environmental factors.

**TABLE 2:** Effects of nitrogen addition and altered precipitation on soil environment factors and plant properties

Treatment	Soil temperature (°C)	Soil moisture (%)	Soil pH	Soil NH_4_^+^‐N (mg/kg)	Soil NO_3_^—^N (mg/kg)	AGB (g/m^2^)	BGB (g/m^2^)
NP(control)	12.84 ± 0.24ab	21.58 ± 0.81b	8.20 ± 0.03a	20.53 ± 1.82b	41.64 ± 2.96d	117.31 ± 15.97c	265.72 ± 27.73c
NP−	13.08 ± 0.30ab	18.86 ± 0.35c	8.22 ± 0.06a	14.33 ± 0.53c	48.24 ± 2.92 cd	107.25 ± 4.31c	221.54 ± 23.77c
NP+	13.08 ± 0.31ab	23.55 ± 0.67a	8.15 ± 0.04ab	20.38 ± 0.69b	42.25 ± 3.16d	136.00 ± 4.75bc	296.77 ± 20.71bc
N + P	12.34 ± 0.13b	21.65 ± 0.45b	8.08 ± 0.02bc	21.65 ± 1.11ab	60.26 ± 4.53ab	161.67 ± 17.10bc	459.99 ± 81.96a
N + P−	13.12 ± 0.24a	18.24 ± 0.17c	7.99 ± 0.04c	19.17 ± 0.91b	68.40 ± 4.89a	182.67 ± 12.68b	422.97 ± 45.25ab
N + P+	12.34 ± 0.11b	24.81 ± 0.21a	8.13 ± 0.02ab	23.92 ± 0.67a	53.91 ± 3.27bc	253.54 ± 42.30a	421.97 ± 28.95ab
*Two‐way ANOVA*							
N	0.169	0.714	**0.013**	**0.036**	**0.000**	**0.018**	**0.018**
P	**0.021**	**0.000**	0.631	**0.003**	0.100	**0.008**	0.585
N × P	**0.021**	0.293	0.048	0.072	0.562	0.145	0.883
Block	0.268	0.773	0.577	0.601	0.690	0.361	0.665

Note

N: nitrogen treatment; P: altered precipitation treatment; N × P: combination of nitrogen addition and altered precipitation. AGB: aboveground biomass; BGB: belowground biomass. Data are represented by mean ± SE (*n* = 5). Bold values indicate that treatment effects are significant (*p* < .05). Different lowercase letters indicate significant difference (*p* < .05). The block was used as a random factor in the two‐way ANOVA.

### 3.2 Responses of N_2_O flux and functional genes to nitrogen addition and altered precipitation

**FIGURE 2:**
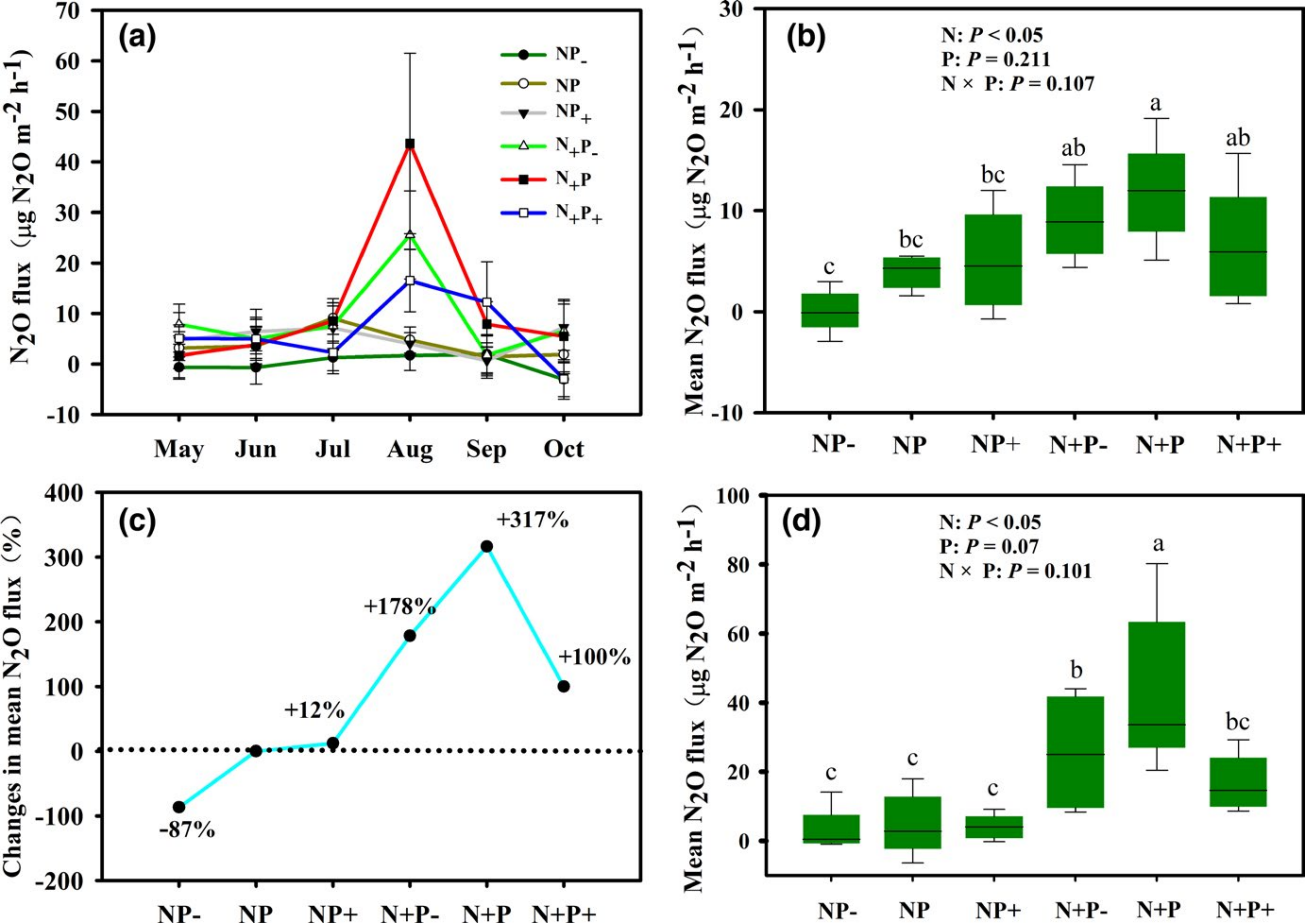
(a) Seasonal dynamics of N_2_O flux and (b) seasonal average N_2_O flux under different conditions. (c) Changes in seasonal average N_2_O flux (compared with control) under different treatments. (d) Average N_2_O flux during the peak period of plant growth. N: nitrogen treatment; P: altered precipitation treatment; N × P: combination of nitrogen addition and altered precipitation. Different letters indicate a significant difference (*p* < .05). Error bars represent the standard error

N_2_O emissions showed a significant seasonal dynamic, with the maximum flux appearing in August (Figure 2a). Although the average flux was relatively small, the alpine steppe presented as the source of N_2_O (*F* > 0) during the growing season under different treatment conditions (Figure 2b). The addition of N resulted in a significant increase in N_2_O emissions (317%) (Figure 2b,c). However, N_2_O emissions were not significantly influenced by altered precipitation or the interaction between N addition and precipitation changes (Figure 2b). To a certain extent, the coupling of N and water alleviated the effect of N input on N_2_O emissions (178% and 100% vs 317%) (Figure 2b,c). Similarly, N_2_O flux during the emission peak was only affected by N addition (Figure 2d).

**FIGURE 3:**
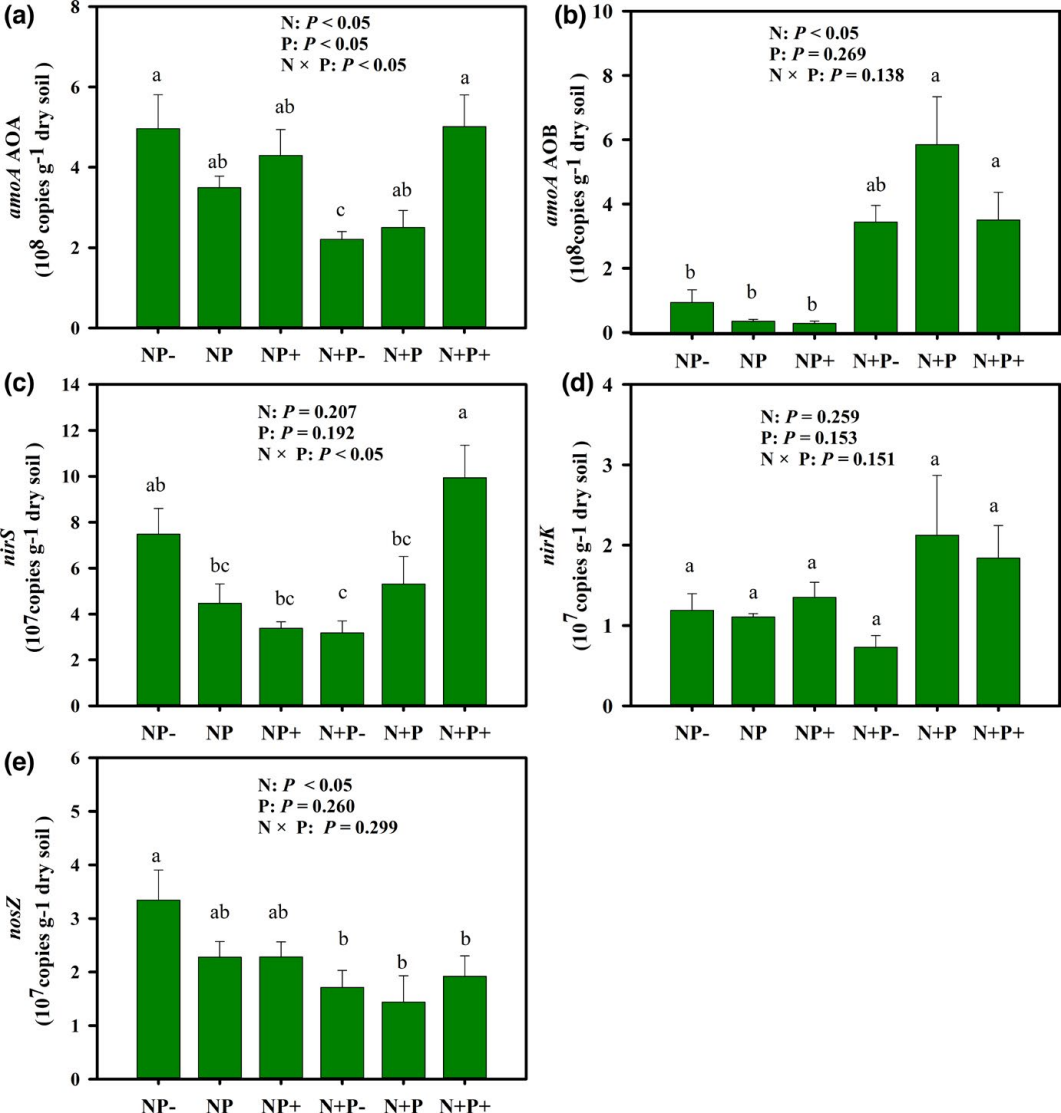
Effects of nitrogen addition and altered precipitation on abundances of functional genes. (a) ammonia‐oxidizing archaea (AOA); (b) ammonia‐oxidizing bacteria (AOB); (c) *nirS*; (d) *nirK*; (E) *nosZ*. N: nitrogen treatment; P: altered precipitation treatment; N × P: combination of nitrogen addition and altered precipitation. Different letters indicate a significant difference (*p* < .05). Error bars represent the standard errors of the means (*n* = 5)

The *amo*A gene abundance of the nitrifier AOA was significantly affected by both N supply and precipitation changes (Figure 3a). However, the AOB *amo*A gene abundance was significantly elevated only by N supply (Figure 3b). Although the denitrifier *nirS* and *nirK* genes regulate the same step in denitrification (nitrite reduction: NO_2_^−^→ NO), only the *nirS* gene abundance was significantly affected by the interaction of N addition and precipitation changes (Figure 3c). The abundance of the *nirK* gene did not significantly differ among the treatments (Figure 2d). The abundance of the *nosZ* gene was reduced under N addition, and altered precipitation did not significantly affect the *nosZ* gene abundance (Figure 3e).

### 3.3 Underlying biotic and abiotic mechanisms related to N_2_O emissions

**FIGURE 4:**
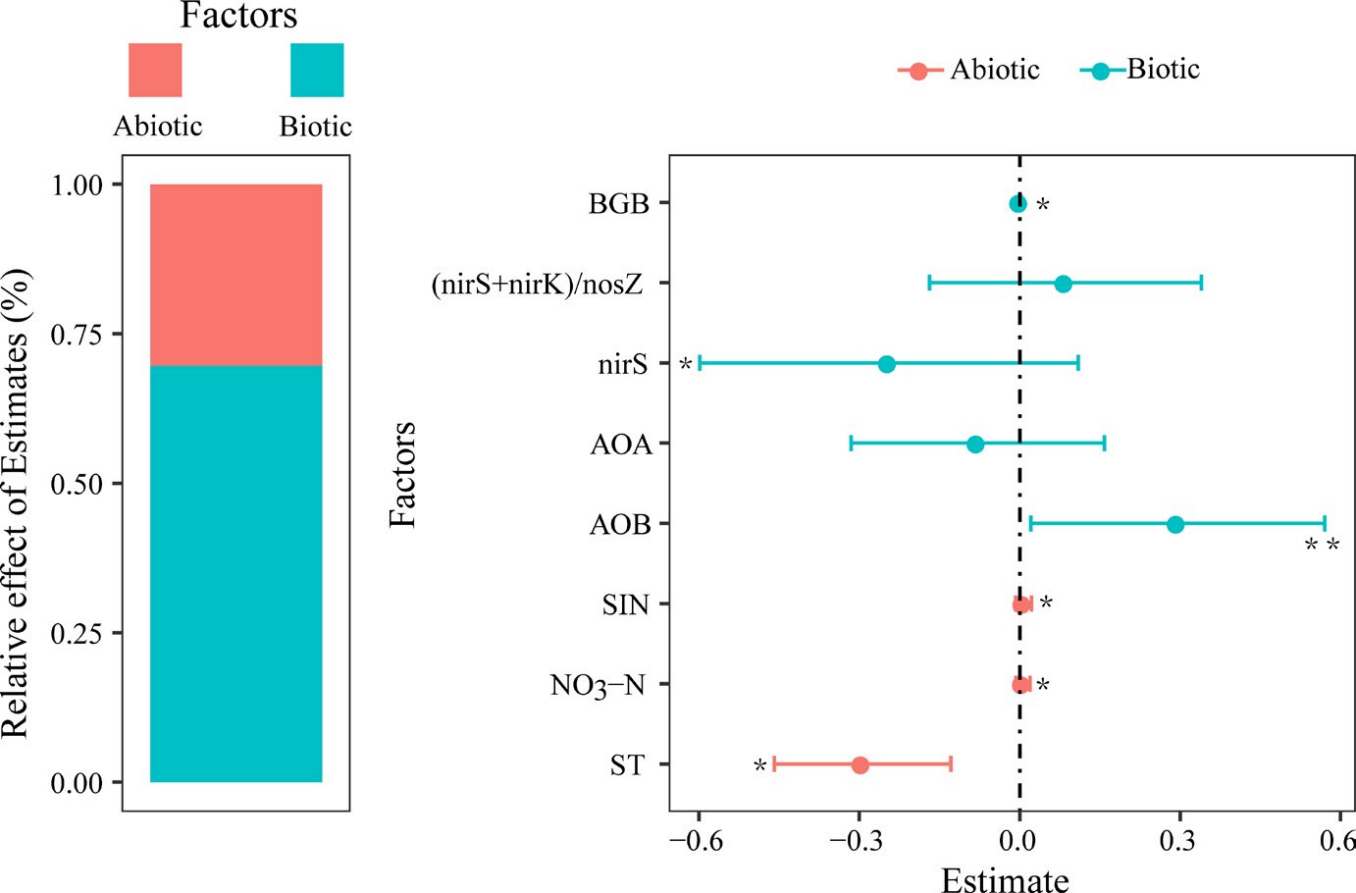
Effects of biotic and abiotic factors on N_2_O flux. AOA: ammonia‐oxidizing archaea; AOB: ammonia‐oxidizing bacteria; *nirS*, *nirK*, and *nosZ*: denitrifying microorganisms; (*nirS* + nirK)/*nosZ*: changes in community structure of denitrifying microorganisms; BGB: belowground biomass; SIN: soil inorganic N (NH_4_^+^‐N and NO_3_^−^‐N); ST: soil temperature. *p < .05; **p < .01

The difference between different regression models was small (see AICc values), and therefore, the significant variables in all models could be used as predictors of N_2_O emissions (Table 3). Multiple regression analysis then identified AOA, AOB, *nirS*, (*nirS* + nirK)/*nosZ*, BGB, SIN, NO_3_^−^‐N, and soil temperature as key predictors of N_2_O emissions (Table 3). Notably, soil moisture was not included in any of the regression models. Furthermore, the VPA showed that biotic factors (especially AOB, nirS, and BGB) had the greatest effect on N_2_O flux (Figure 4). Of the abiotic factors analyzed, the effect of soil temperature on N_2_O flux was greater than that of substrate concentration (Figure 4), likely because the ecosystem was N‐saturated.

**TABLE 3:** Results of multiple regression analysis examining the factors regulating N_2_O emissions

Models	Intercept	Factors								
		Biotic					Abiotic			AICc
		AOA	AOB	*nirS*	(*nirS* + nirK)/*nosZ*	BGB	SIN	NO_3_^−^‐N	ST	
1	−2.56					0.0003		0.03	−0.31	74.4
2	9.63		0.57	−0.54					−0.34	74.6
3	−2.92					0.0002	0.03		−0.31	75.1
4	12.3	−0.62	0.44						−0.3	75.2
5	−2.74								−0.32	76.2
6	13.14		0.49							76.2
7	8.91		0.44	−0.79	0.71	0.0002	0.04		−0.35	76.3
8	11.8		0.49	−0.53					−0.26	76.3
9	0.01		0.48	−0.69	0.42				−0.34	76.3

Note

AOA, ammonia‐oxidizing archaea; AOB, ammonia‐oxidizing bacteria; *nirS*, *nirK*, and *nosZ*, denitrifying microorganisms; (*nirS*+nirK)/*nosZ*, changes in community structure of denitrifying microorganisms; BGB, belowground biomass; SIN, soil inorganic N (NH_4_^+^‐N and NO_3_^−^‐N); ST, soil temperature; AICc, Akaike information criterion. Models were sorted by AICc.

**FIGURE 5:**
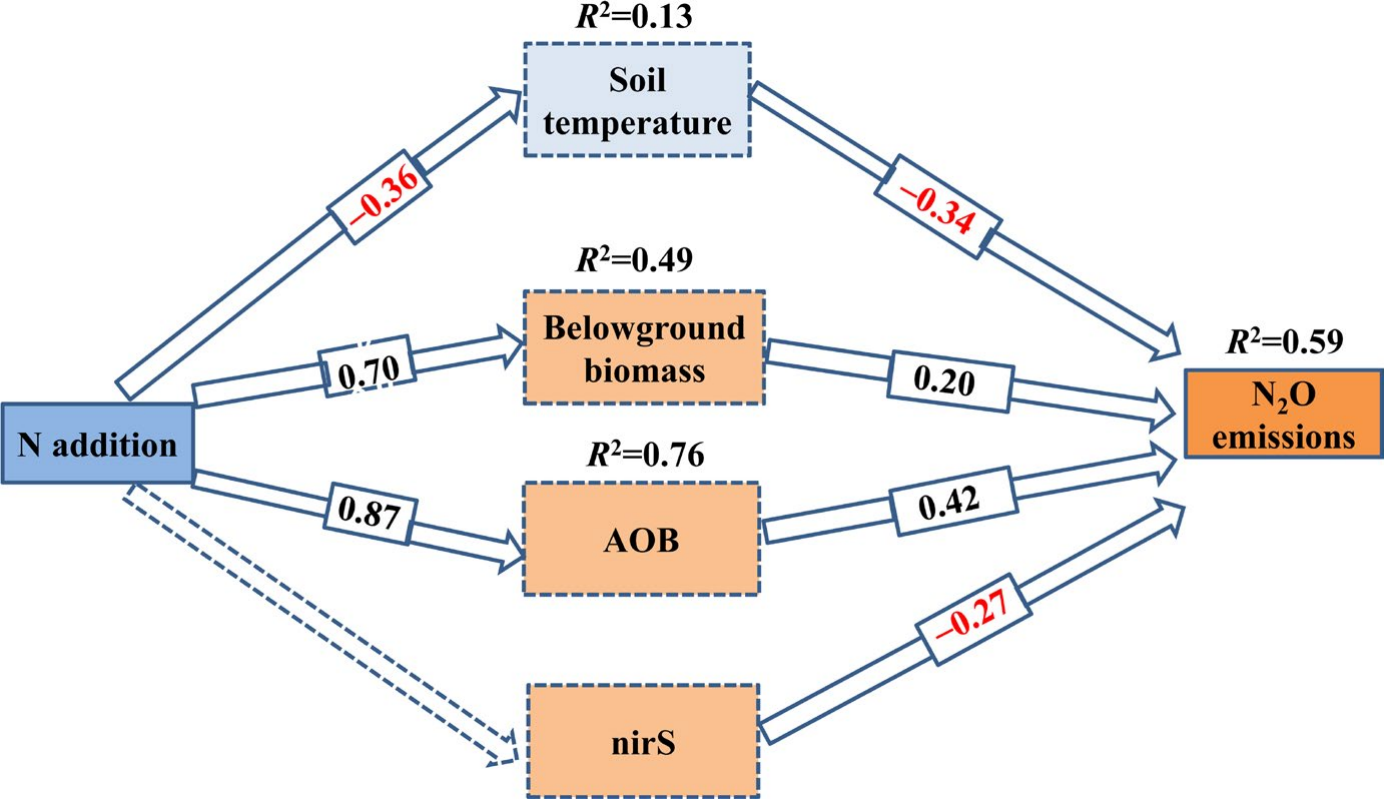
Structural equation model (SEM) analysis examining the effects of N addition on biotic and abiotic properties and N_2_O emissions. AOB: ammonia‐oxidizing bacteria; *nirS*: denitrifying microorganisms. Values on arrows represent standardized path coefficients. The marked red values represent significant negative effects. The dashed line means no significant effect. Results of model fitting: χ^2^ = 0.18, *p* = .23, RMSE = .12

Key factors (AOB, *nirS*, and ST) that showed strong and significant effects on N_2_O flux in the VPA were considered in the SEM. Considering that BGB was an important feature of plant attributes, it was also included in the SEM. SEM analysis showed that both biotic and abiotic factors played a role in regulating N_2_O emissions, and they explained 59% of the variation in N_2_O emissions in the ecosystem (Figure 5). An increase in the abundance of AOB and BGB, caused by N addition, directly promoted N_2_O emissions (Figure 5). However, soil temperature and *nirS* had significant negative effects on N_2_O emissions (Figure 5).

## 4 DISCUSSION

### 4.1 Effect of long‐term nitrogen addition on N_2_O emissions

The results from our field experiments show that the alpine steppe was a net source of N_2_O. N addition significantly increased N_2_O emissions (Figure 2). Most terrestrial ecosystems, especially grassland ecosystems, are widely limited by N (Geng et al., 2019; Lu et al., 2011). N enrichment increases N available in soil, even reaching N saturation, and available N directly affects N_2_O emissions (Peng et al., 2018). In arid and semi‐arid grassland ecosystems, nitrification is usually the predominant cause of N_2_O emissions (Zhang et al., 2020). Nitrification is the process of converting NH_4_^+^‐N into NO_3_^−^‐N, which forms N_2_O, thus enhancing N_2_O emissions (Li et al., 2020). We found that N addition (NH_4_^+^‐N: NO_3_^−^‐N; 1:1) significantly increased the inorganic N content in soil (Table 2). NO_3_^−^‐N was present at a much higher concentration than NH_4_^+^‐N (Table 2), indicating that nitrification may be the predominant pathway of N_2_O emissions in this alpine steppe. NH_4_^+^‐N is the key substrate for nitrification. However, we found that NH_4_^+^‐N had no significant effect on N_2_O emissions. A possible explanation for this finding is that the ecosystem is already N‐saturated at the current N addition level (10 g m^−2^ year^−1^) (Peng et al., 2018).

We also found that changes in abiotic factors and plant attributes caused by N addition regulated N_2_O emissions (Figure 5). Generally, soil N cycling largely depends on soil temperature in alpine ecosystems. In particular, warming was found to drive N_2_O production and emissions (Griffis et al., 2017). In contrast, Zhang et al. (2020) pointed out that warming did not significantly boost N_2_O emissions. However, rising temperatures negatively affected N_2_O emissions in our study (Figures 4, 5). It is possible that higher temperatures aggravate evapotranspiration and decrease soil water availability, thereby limiting various microbial N cycling processes (Shi et al., 2012). Overall, arid soils are detrimental to the abundance and activity of N cycling microbiomes (Waghmode et al., 2018). Therefore, warming may induce more negative effects than positive effects on soil N cycling. Considering the high sensitivity of alpine regions to global climate change and that small temperature changes may have different effects on soil N cycling, future research should focus more on the effects of warming on ecosystems. It is worth noting that plant biomass is also a key driver of N_2_O emissions (Figure 4). N input facilitated plant growth, especially root growth (Table 2). Soil labile C via root secretion may accelerate N_2_O emissions because denitrification is commonly driven by high available C as a source of energy (Li et al., 2020). This phenomenon is consistent with our conclusion that the increase in belowground biomass enhanced N_2_O emissions (Figure 5).

The increase in N_2_O emissions may also be ascribed to functional microbes (Figure 5). The community composition and diversity of N cycling microbes are directly involved in N_2_O production and emissions. Microbial functional genes associated with N cycling encode some key oxidoreductases and are therefore used as genetic markers for nitrifying and denitrifying microorganisms (Mushinski et al., 2021). The functional genes of AOA and AOB usually regulate the rate‐limiting step (ammonia oxidation: NH_3_ → NH_2_OH) in nitrification (Hu et al., 2015; Lu et al., 2015). Some studies have indicated that N_2_O emissions were promoted by increased abundances of both AOA and AOB (Brin et al., 2019; Linton et al., 2020). However, we found that N addition only significantly increased the abundance of AOB (Figure 3), and the functional genes of AOB rather than those of AOA dominated the N_2_O emissions from nitrification (Figure 5). Di et al. (2009) also showed that N_2_O emissions are driven by AOB and not AOA in N‐enriched grassland ecosystems. Previous investigations demonstrated that AOA and AOB occupy different niches (Nicol et al., 2008). AOA and AOB play a dominant role in acidic and alkaline soils, respectively, and pH is the chief factor for niche separation (Hu et al., 2015; Tzanakakis et al., 2019). The alkaline conditions (pH > 7.5; Table 2) in this study may be more conducive to the activity of AOB, which further supports our conclusion that AOB controlled the N_2_O emissions in nitrification. For the denitrifiers related to denitrification, the key step of denitrification (NO_2_^−^ → NO→ N_2_O) is generally mediated by *nirS*‐ or *nirK*‐encoding nitrite reductase (Butterbach‐Bahl et al., 2013). Oppositely, the nitrous oxide reductase encoded by *nosZ* promotes N_2_O reduction (N_2_O→ sN_2_), thereby reducing N_2_O emissions (Butterbach‐Bahl et al., 2013; Hu et al., 2015). In this study, N addition did not significantly affect abundance of *nirS* and *nirK*, but significantly decreased abundances of *nosZ* (Figure 3). Decreased *nosZ* abundance is unfavorable to the reduction of N_2_O, thus aggravating N_2_O emissions (Bowen et al., 2020). Previous studies have also shown that high (*nirS*+*nirK*)/*nosZ* ratios result in a strong N_2_O emission capacity (Hu et al., 2015; Yang et al., 2018). However, we found that the (*nirS* + *nirK*)/*nosZ* ratio had little effect on N_2_O emissions (Figure 4). As our study area is part of a semi‐arid ecosystem, the denitrifying communities involved in N_2_O production are more likely to be inhibited by aerobic conditions in arid soils (Waghmode et al., 2018). However, denitrification can also be driven by fungal pathways (heterotrophic denitrification) in semi‐arid ecosystems (Crenshaw et al., 2007). Therefore, more studies are required to increase our understanding of denitrifying pathways for N_2_O emissions in semi‐arid areas.

### 4.2 Effects of long‐term altered precipitation and its interaction with nitrogen addition on N_2_O emissions

Changed precipitation regimes also play an important role in modulating soil N cycling (Chen et al., 2013; Cregger et al., 2014; Lin et al., 2016). Li et al. (2020) demonstrated that increased precipitation exacerbated N_2_O emissions in grassland ecosystems while reductions in precipitation mitigated N_2_O emissions. In this study, however, we observed that altered precipitation patterns did not affect N_2_O emissions (Figure 2), indicating that precipitation is not the major factor of N_2_O emissions. On the one hand, water addition may diminish soil N pools (soil inorganic N) by promoting plant N uptake and soil leaching, neither of which are conducive to nitrification and denitrification (Austin et al., 2004; Kruger et al., 2021; Lin et al., 2016). On the other hand, water reduction (i.e., prolonged drought treatment) had little effect on N_2_O emissions, possibly because the alpine steppe itself belongs to a semi‐arid grassland ecosystem and is insensitive to drought treatment (Dijkstra et al., 2013). In addition to the amount of precipitation, altered precipitation regimes are also characterized by changes in precipitation frequency, which may lead to unexpected consequences in semi‐arid ecosystems (Shi et al., 2021). Thus, an evaluation of the effects of changes in rainfall frequency on N_2_O emissions is urgently required.

Climate change involves multiple elements, including the co‐occurrence of precipitation with N deposition (Rillig et al., 2019). The interaction between altered precipitation regimes and N addition did not significantly affect N_2_O emissions in our experiment (Figure 2). There are several mechanisms that could contribute to this finding. Ordinarily, N and water co‐limitation is a typical feature of arid grassland ecosystems (Austin et al., 2004; Lü and Han, 2009). The responses of grassland ecosystems to N deposition are strongly regulated by precipitation patterns (Harpole et al., 2007). Increased precipitation, particularly under the background of N addition, could increase plant access to soil inorganic N resources (Li et al., 2019), so the effect of N addition on N_2_O emissions may be alleviated by water addition. In addition, decreased precipitation may suppress microbial activity, leading to inefficient N assimilation, despite the presence of large amounts of N substrates in the soil (Homyak et al., 2017; Li et al., 2020). Overall, the response of N_2_O emissions to the co‐occurrence of precipitation pattern changes and N addition was not obvious. However, a slight trend was observed, indicating that precipitation changes may attenuate the effect of N addition on N_2_O emissions to some extent (Figure 2). Hence, the interplay of altered rainfall regimes and N deposition cannot be ignored in future work on N cycling.

## 5 CONCLUSIONS

Our field experiments show that the alpine steppe was a net source of N_2_O. Our results also demonstrate that N addition intensified N_2_O emissions, while altered precipitation and its interaction with N addition did not affect N_2_O emissions. Nitrification rather than denitrification dominated N_2_O emissions. Changes in N_2_O flux were attributable to the synergy between functional microorganisms and soil abiotic parameters. The abundance of AOB was responsible for N_2_O emissions due to nitrification. Additionally, plant attributes (belowground biomass) and abiotic soil factors (soil temperature) were primary predictors of N_2_O emissions. This study provides necessary insight to predict the future responses of N_2_O emissions to long‐term N deposition and precipitation alterations in alpine grasslands.

## CONFLICT OF INTEREST

The authors declare no competing financial interests.

## AUTHOR CONTRIBUTION

**Yang Yang:** Data curation (lead); Formal analysis (lead); Investigation (lead); Writing‐original draft (lead); Writing‐review & editing (lead). **Yuanming Xiao:** Data curation (equal); Formal analysis (equal); Investigation (equal). **Changbin Li:** Data curation (equal). **Bo Wang:** Writing‐original draft (equal). **Yongheng Gao:** Writing‐original draft (equal). **Guoying Zhou:** Funding acquisition (lead); Investigation (equal); Project administration (lead); Writing‐original draft (equal); Writing‐review & editing (equal).

## Data Availability

We had uploaded our data to the Dryad. https://doi.org/10.5061/dryad.fj6q573vz
